# Adipose tissue macrophages: going off track during obesity

**DOI:** 10.1007/s00125-016-3904-9

**Published:** 2016-03-03

**Authors:** Lily Boutens, Rinke Stienstra

**Affiliations:** Department of Medicine, Radboud University Medical Center, Nijmegen, the Netherlands; Nutrition, Metabolism and Genomics Group, Wageningen University, Bomenweg 2, 6703 HD Wageningen, the Netherlands

**Keywords:** Adipose tissue, Fat, Immune cells, Inflammation, Insulin resistance, Macrophages, Metabolism, Obesity, Review

## Abstract

Inflammation originating from the adipose tissue is considered to be one of the main driving forces for the development of insulin resistance and type 2 diabetes in obese individuals. Although a plethora of different immune cells shapes adipose tissue inflammation, this review is specifically focused on the contribution of macrophages that reside in adipose tissue in lean and obese conditions. Both conventional and tissue-specific functions of adipose tissue macrophages (ATMs) in lean and obese adipose tissue are discussed and linked with metabolic and inflammatory changes that occur during the development of obesity. Furthermore, we will address various circulating and adipose tissue-derived triggers that may be involved in shaping the ATM phenotype and underlie ATM function in lean and obese conditions. Finally, we will highlight how these changes affect adipose tissue inflammation and may be targeted for therapeutic interventions to improve insulin sensitivity in obese individuals.

**Table:** 

**Highlights**
• Macrophages play a significant role in regulating adipose tissue functioning during health and disease
• In addition to conventional functions such as clearing cellular debris and participating in tissue immune surveillance, lipid buffering is an important function of ATMs
• Obesity-induced inflammation, characterised by an elevated number of proinflammatory macrophages in adipose tissue, has been suggested to contribute to systemic insulin resistance
• Their origin, as well as a combination of peripheral changes and adipose tissue-derived stressors, probably contribute to ATM dysfunction and inflammatory traits during obesity
• Identification of transcriptional differences between ATMs from lean vs obese adipose tissue at several key points during the development of obesity and insulin resistance may reveal upstream triggers, regulatory factors and intracellular pathways that shape ATM function
• Targeting metabolic capacity rather than the inflammatory phenotype of ATMs may hold potential to restore ATM function and adipose tissue homeostasis in obese individuals

## Introduction

Historically, adipose tissue is known to perform a number of functions including storage of excess energy, cold insulation and protection of vital organs. Intense research efforts over the last decades have greatly furthered our understanding of adipose tissue biology and led to the identification of various additional functions. The adipose tissue—composed of adipocytes and non-adipocyte cells, including endothelial cells, pre-adipocytes and various types of immune cells [1]—is known to produce and secrete a wide variety of so-called adipokines. The release of adipokines enables communication with other cells and tissues throughout the body involved in the regulation of energy metabolism, satiety and various other processes.

The central role of adipose tissue in homeostasis becomes apparent during the development of obesity, which is a major risk factor for the development of insulin resistance [2]. During the development of obesity, storage of excess amounts of triacylglycerol in adipose tissue is associated with altered release of adipokines and cytokines that control local and systemic inflammatory processes and interfere with insulin signalling [3]. At the cellular level, it has been well established that obesity promotes robust changes in adipose tissue morphology [4]. In particular, hypertrophy of adipocytes and significant alterations in the number and composition of immune cells promote a state of chronic low-grade inflammation during obesity that is strongly associated with the development of insulin resistance and type 2 diabetes [1].

Macrophages are immune cells that have gained much attention as important contributors to adipose tissue functioning. Whereas macrophages in lean mice and humans make up around 5% of the cells in adipose tissue, during obesity they constitute up to 50% of all adipose tissue cells [5]. As well as increasing in number, adipose tissue macrophages (ATMs) change their localisation and inflammatory features during obesity. Contrary to the lean state, in which ATMs are distributed throughout the adipose tissue exposing limited inflammatory properties, ATMs in obese adipose tissue are located around dead adipocytes and form so-called crown-like structures (CLSs) while displaying profound proinflammatory features [6–8]. Macrophage presence in CLSs within obese adipose tissue has been directly linked with insulin resistance [9, 10]. Notably, however, the importance of macrophage-mediated inflammation in determining insulin resistance is related to long-term exposure to a high-fat diet (HFD) whereas the initial stage of insulin resistance is independent of macrophage action [11].

In contrast to mice, inflammatory changes in human adipose tissue during obesity are somewhat less pronounced, although the presence of CLSs is positively correlated with a worsening in insulin resistance levels [12]. Furthermore, inter-individual differences in the degree of adipose tissue inflammation are likely to exist in humans and the importance of macrophage-mediated adipose tissue inflammation on the development of insulin resistance will vary between obese individuals.

In addition to macrophages, numerous other types of immune cell populate the adipose tissue and affect its function [5, 13]. Indeed, dendritic cells [14], mast cells [15], neutrophils [16], B cells [17, 18] and T cells [19–21] have been found to reside in adipose tissue during obesity and contribute to the development of adipose tissue inflammation and insulin resistance. In particular, neutrophils and CD8^+^ T cells have recently gained considerable attention because of the observed early influx into adipose tissue upon HFD-feeding and their potential contribution either to attracting macrophages or affecting their phenotype [16, 19, 22]. On the other hand, forkhead box (FOX)P^+^ regulatory CD4^+^ T cells have been found to reduce in number prior to the accumulation of macrophages in obese adipose tissue [23]. In line with their well established role in dampening proinflammatory signalling, reduced presence of regulatory CD4^+^ T cells in adipose tissue might explain the accumulation and/or proinflammatory signalling of ATMs during obesity. Overall, these findings suggest a strong interplay between adaptive and innate immunity that together determine the inflammatory characteristics of obese adipose tissue. An excellent overview of the contribution of various immune cells to adipose tissue biology can be found elsewhere [24, 25]. This review will primarily focus on the function of macrophages in adipose tissue and their contribution to the inflammatory traits of obese adipose tissue.

Macrophages are unique in their capacity to quickly adapt to a changing environment, causing them to embrace a variety of phenotypes ranging from anti-inflammatory to proinflammatory. In virtually every tissue, macrophages are actively involved in maintaining tissue homeostasis by clearing cellular debris, participating in tissue immune surveillance and resolving inflammation [26]. Although ATMs have been extensively associated with the development of obesity and adipose tissue inflammation, they are known to populate lean adipose tissue as well, executing numerous functions crucial for maintaining adipose tissue homeostasis [27].

In this review, we will touch upon both well established and recently identified functions of macrophages in lean and obese adipose tissue. The origin of macrophages residing in lean and obese adipose tissue will be addressed and several triggers that may account for an increase in number and a phenotypical switch of ATMs during the development of obesity will be discussed. Finally, this knowledge is used to pinpoint relevant future research directions to establish potential therapeutic targets for shifting macrophage phenotype and function in order to promote adipose tissue health.

## What are the functions of macrophages in adipose tissue?

### ATMs and efferocytosis

In 1908, Elie Metchnikoff received the Nobel Prize for his important discovery of the phagocytic activity of macrophages. Nowadays phagocytosis is still considered to be the most prominent function of macrophages. Although Metchnikoff specifically described the engulfment of microbes, phagocytosis also encompasses the engulfment of apoptotic endogenous cells in a process termed ‘efferocytosis’ [28]. Efficient efferocytosis licenses anti-inflammatory removal of aged or damaged cells by macrophages and is crucial to maintain homeostasis in tissues where cellular turnover occurs [28, 29]. Cellular turnover is also active in adipose tissue, characterised by continuous removal of adipocytes and their replacement by new adipocytes. Depending on the study population, the adipose tissue depot studied and the methodology used, calculations of the rate of adipocyte turnover in adults range from 10% per year [30] to up to 58–106% per year [31]. Since the total number of adipocytes in adipose tissue is thought to be set during childhood and thus to remain stable in adults [30, 32], probably a continuous cycle of cell death and replenishment exists in which macrophages presumably play a key role. Indeed, studies in which excessive adipocyte death is induced through activation of caspase 8 [33] or ongoing stimulation of lipolysis [34] have revealed anti-inflammatory macrophage accumulation around dead adipocytes, pointing to the need for macrophages during removal of dead adipocytes and the capacity of ATMs to clear high numbers of dead adipocytes while maintaining an anti-inflammatory state . The importance of efficient efferocytosis becomes apparent in several chronic inflammatory diseases, such as cystic fibrosis, chronic obstructive pulmonary disease and asthma, where ineffective clearance of dead cells has been identified as important source of proinflammatory signalling [29]. In obese adipose tissue, macrophages surrounding dead adipocytes in CLSs demonstrate proinflammatory features [6–8]. Since efficient efferocytosis is known to be accompanied by an anti-inflammatory macrophage trait, the presence of proinflammatory ATMs surrounding dead adipocytes in the obese state might reflect futile adipocyte clearance contributing to adipose tissue inflammation.

### ATMs, adipocyte lipolysis and lipid buffering

Building upon their unique role as professional phagocytes, recent data suggest that macrophages facilitate acute metabolic tasks executed by the adipose tissue. During lipolysis induced by either fasting or pharmacologic adrenergic activation, macrophages rapidly infiltrate the adipose tissue and adopt an anti-inflammatory phenotype [35]. In adipose tissue, macrophages buffer lipolysis by taking up and storing excessive amounts of adipocyte-released lipids, supervising gradual lipid release into the bloodstream [35–37]. Recently, macrophages have also been linked to controlling adipocyte lipolysis during cold exposure. Upon exposure to cold, anti-inflammatory macrophages have been reported to secrete catecholamines to stimulate adipocyte lipolysis in both inguinal and brown adipose tissue [36, 38–40]. These acute challenges appear to promote macrophage mobility and quick adaptation to a changing environment facilitating tissue flexibility.

Interestingly, 7 days of continuous infusion of a β3-adrenergic receptor agonist to induce adipocyte lipolysis not only results in an influx of anti-inflammatory macrophages that adopt a lipid-laden cell appearance, it also increases the presence of dead adipocytes and the formation of CLSs. Within these CLSs, anti-inflammatory macrophages have been found to recruit and stimulate platelet-derived growth factor receptor (PDGFR)α^+^ adipocyte progenitors to differentiate [34]. Intriguingly, these adipogenic clusters near CLSs point to a direct link between efferocytosis, lipid buffering and adipose tissue remodelling by macrophages in lean adipose tissue.

In obese adipose tissue, macrophages surrounding dead adipocytes in CLSs also form multiple intracellular lipid droplets and activate transcriptional programs involved in lysosomal lipolysis [41]. The presence of lipid-filled macrophages in CLSs of obese adipose tissue in both humans [42] and mice [8] is indicative of macrophages attempting to buffer and process excessive amounts of lipids originating from adipocytes in a similar fashion to that observed in lean adipose tissue. However, increased spilling of NEFAs during obesity is suggestive of unsuccessful lipid buffering by ATMs. Thus, although engulfment and storage of lipids by ATMs seems to hold functional relevance in both lean and obese adipose tissue, in obesity the lipid buffering capacity of ATMs appears to be insufficient.

### ATMs and adipogenesis

The close correlation between adipocyte death and new cell formation through adipogenesis that has been observed in lean adipose tissue may not hold true during obesity. While macrophages in obese adipose tissue have been found to populate adipogenic clusters and facilitate angiogenesis and adipogenesis, adipogenic clusters are formed at sites away from CLSs [34, 43, 44]. This might relate to the proinflammatory state of macrophages in CLSs in obese adipose tissue. Although it has been proposed that proinflammatory signalling is needed for efficient adipogenesis to occur [45], multiple cell culture experiments have demonstrated that proinflammatory macrophages inhibit both proliferation and differentiation of adipogenitor cells [46–49]. The proinflammatory phenotype of macrophages in CLSs of obese adipose tissue may suppress adipogenesis and thus explain the appearance of adipogenic clusters away from inflammatory CLSs [34].

In contrast to studies postulating a role for macrophages in adipogenesis, other studies have argued that ATMs inhibit adipogenesis in obese adipose tissue [49, 50]. For example, it was found that exposure of adipocyte progenitors to conditioned medium of CD14^+^ cells from obese adipose tissue blocks adipogenesis [49]. A prominent factor involved in suppressing adipogenesis is Wnt-5a, which is abundantly expressed in ATMs and circulating monocytes from individuals with obesity and type 2 diabetes compared with lean individuals [50]. Overall, the effect of macrophages on adipogenesis in obese adipose tissue is still being debated. One might hypothesise that the role of macrophages shifts during the progression of obesity from a predominant role in stimulating adipogenesis at the start of adipose tissue expansion to inhibiting adipogenesis once obesity progresses. The observation that adipogenic clusters containing macrophages appear at an early stage of adipose tissue expansion during obesity, yet decline in number upon the progression of obesity, supports this hypothesis [43]. Reduced adipogenesis at later stages of obesity would promote hypertrophy of adipocytes to allow for storage of the excess amounts of lipids entering the adipose tissue, which is in turn linked to metabolic dysfunction such as adipocyte insulin resistance [51, 52]. Indeed, increased adipocyte size has been found to correlate with macrophage presence in obese adipose tissue [53, 54]. However, future in vivo studies will be needed to further unravel the role of ATMs in adipogenesis and adipocyte hypertrophy in both lean and obese adipose tissue. In conclusion, multiple functions of macrophages in lean adipose tissue, including the prevention of lipid spillover into the circulation and licensing adipose tissue remodelling, appear to be impaired in obese adipose tissue. It is likely that impairment of these functions executed by ATMs contributes to loss of adipose tissue homeostasis in obese individuals. Differential metabolic and inflammatory traits of ATMs residing in either lean or obese adipose tissue may underlie shifts in its functional output and thus total adipose tissue functioning. Hence, careful identification of ATM phenotypes in lean and obese individuals might provide evidence for functional rewiring of ATMs upon the development of obesity.

## What are the effects of the adipose tissue environment on macrophage phenotype?

### Macrophage phenotype in lean vs obese adipose tissue

Besides increasing in number, ATMs are known to dramatically change their phenotype during obesity. It has been suggested that the majority of the macrophage population in adipose tissue of obese mice consists of F4/80^+^CD11c^+^ or ‘classically activated’ M1 macrophages, characterised by increased expression levels of TNFα and inducible nitric oxide synthase (iNOS) [8]. By contrast, most of the macrophages in lean adipose tissue can be identified as F4/80^+^CD206^+^CD301^+^CD11c^−^ macrophages, resembling an alternatively activated M2 phenotype that is characterised by the expression of genes encoding anti-inflammatory proteins including Ym1, arginase 1 and IL-10 [8, 27]. A balanced ‘M0’ F4/80^+^CD206^−^CD11c^−^ phenotype has also been reported [55], supporting an even greater diversity of inflammatory macrophage subtypes in the adipose tissue. However, in light of the metabolic functions performed by ATMs, a classification based on their inflammatory properties may not suffice. In line with this hypothesis, both M1 and M2 markers have been identified on a phenotypical heterogeneous population of macrophages in obese adipose tissue [56]. Interestingly, a metabolic classification has recently been put forward, characterised by increased lysosomal biogenesis and subsequent lipid catabolism as a hallmark of ATMs in obese adipose tissue [41]. Rather than cytokine-driven proinflammatory activation of ATMs, fatty acids (FAs) are the main trigger for metabolic activation of ATMs in obese adipose tissue. Importantly, metabolic changes in lipid metabolism may predominantly underlie inflammatory activation of macrophages in adipose tissue, promoting an inflammatory phenotype of ATMs that may not fit into any of the classically defined inflammatory macrophage subtypes [57].

### Macrophages in human vs mouse adipose tissue

In parallel with an increase in ATMs during obesity in mice, macrophage numbers have also been found to increase in human obese adipose tissue [5, 7]. In contrast to the proinflammatory phenotype of macrophages found in many animal studies, an ‘M2-type’ macrophage with remodelling capacity, yet still capable of secreting substantial quantities of proinflammatory cytokines, has been identified in adipose tissue of obese individuals [58]. The mixed inflammatory phenotype of ATMs in obese individuals is further illustrated by the presence of both CD206 and CD11c on the ATM membrane, markers generally used to distinguish between M2- and M1-type macrophages [59]. Despite their anti-inflammatory characteristics, this population of CD11c^+^CD206^+^ macrophages has been associated with insulin resistance [60]. Interestingly, the M2 macrophage marker CD163 has even been proposed to be the single macrophage marker that significantly correlates with HOMA-IR [61]. As mentioned before, less pronounced correlations between ATM numbers and insulin resistance have been found in humans, compared with mice [62–64]. It is likely that inter-individual differences in the development of adipose tissue inflammation during obesity exist and contribute to the inconsistency in reported correlations between ATMs, adipose tissue inflammation and the development of insulin resistance in humans.

### Location: visceral vs subcutaneous adipose tissue

Adipose tissue is stored at various locations throughout the body, characterised by specific metabolic and inflammatory properties. Both subcutaneous (scAT) and visceral (vAT) storage depots exist, which are identified based on their anatomical location and differentially contribute to the development of metabolic abnormalities [65]. Epidemiological evidence demonstrates that vAT mass is a dominant risk factor for the development of metabolic abnormalities including insulin resistance [65]. Ex vivo experiments have shown that the inflammatory status, determined by the release of cytokines, is elevated in vAT compared with scAT [66]. This would imply that the number and/or phenotype of macrophages differ between the two adipose tissue depots. Indeed, various studies have shown an enhanced number of macrophages in vAT vs scAT [62, 67]. However, others have failed to report any differences in macrophage cell numbers [66]. In animal studies, bigger adipocytes were found in scAT compared with vAT, which negatively correlated with the number of CLSs in the adipose tissue depots [10]. Moreover, distinct rates of adipocyte turnover, adipocyte lipolysis and blood flow, with consequences for oxygen and nutrient availability, have been detected in scAT vs vAT [68, 69]. Although controversy about the different characteristics exists, distinct microenvironments in the adipose tissue depots might importantly affect macrophage phenotypes. Phenotypical differences in macrophages that populate either vAT or scAT may be exemplified by the higher expression of proinflammatory cytokines by ATMs in vAT [67, 70, 71].

In general, most studies are mainly focused on determining the inflammatory phenotypes of ATMs. However, in line with the metabolic functions executed by ATMs, inflammatory markers may not suffice to distinguish functional from dysfunctional ATMs. It would be interesting to unravel whether macrophages within human adipose tissue display a metabolically activated phenotype, as has been identified recently in mice [57]. Moreover, deciphering what type of metabolic and inflammatory changes in ATMs finally result in functional differences in obese vs lean adipose tissue might improve our understanding of obesity-induced adipose tissue inflammation. Multiple triggers may underlie the macrophage phenotype, explaining the distinct responses of ATMs to several challenges in obese and lean adipose tissue. In order to shed light on such triggers, one should consider how and where the phenotype of the ATMs is being affected.

## From where do ATMs originate?

Contrasting with the prevalent idea that all tissue macrophages are derived from circulating monocytes, novel techniques that allow fate mapping have revealed that not all tissue-resident macrophages originate from monocyte precursors [72]. Many resident tissue macrophages are established during early embryonic development and are maintained during adulthood independently of an influx of blood monocytes [73]. However, inflammation has been shown to drive the recruitment of blood monocyte-derived macrophages that replace embryonically established resident macrophage populations in the myocardium and other tissues [74]. Surprisingly little is known about the origin of adipose tissue-resident macrophages and the maintenance of this population.

### Monocyte recruitment

It is well established that adipocytes are able to produce specific adipokines that function as chemoattractants for circulating monocytes. Monocyte chemoattractant protein-1 (MCP-1) is one such chemokine that is known to be produced in large amounts by adipocytes and is robustly increased in obesity [75]. Indeed, overexpression of MCP-1 in adipose tissue promotes macrophage accumulation [76]. Interestingly, the presence of the C–C chemokine receptor (CCR) type 2, which allows monocytes to respond to MCP-1, is a typical characteristic of newly recruited macrophages that is used to distinguish them from resident macrophages in a variety of tissues [74] including fat [7]. These observations imply that monocyte recruitment through MCP-1 is important in populating adipose tissue with macrophages, and that this pathway is enhanced in the presence of obesity.

In addition to increased secretion of MCP-1 by adipose tissue, obesity is characterised by a significantly increased number of CCR2 molecules on circulating monocytes [77]. Moreover, monocytes from obese individuals demonstrate a higher chemotactic activity, which is associated with both insulin resistance and CCR2 expression [77]. Interestingly, the migratory behaviour itself might be an important influence on macrophage phenotype. This is illustrated by the finding that the inflammatory state is linked to the migratory capacity of macrophages into adipose tissue [6, 8, 78]. In addition to a higher degree of chemotaxis, CCR2^+^ tissue macrophages may have an increased inflammatory phenotype compared with CCR2^−^ tissue macrophages, which is illustrated by an induction of genes involved in the regulating of the NLRP3 inflammasome, at least in the myocardium [74]. The NLRP3 inflammasome allows processing and release of active IL-1β. IL-1β controls inflammatory responses and is involved in the development of insulin resistance [79, 80]. Similar differences appear to exist in ATMs isolated from obese animals shown by enhanced expression of CCR2 and inflammatory cytokines compared with the ATMs of lean animals [7]. However, monocyte recruitment to adipose tissue may not solely depend on MCP-1-controlled pathways, as the absence of MCP-1 does not completely curtail monocyte influx into adipose tissue [81]. Indeed, other chemokine receptor/ligand complexes have been shown to play a role in these processes, including CCR5 [82].

### Bone marrow myelopoiesis

Recent evidence has shed light on mechanisms and signals that have a vital role in controlling the recruitment of monocytes during obesity and go beyond MCP-1 and other chemokine-signalling modules. Indeed, the bone marrow has been identified as an important contributor to the ATM pool [5, 83]. The development of obesity has been found to stimulate bone marrow myelopoiesis, which licenses the ongoing infiltration of monocytes into adipose tissue [84, 85]. Interestingly, it appears that signals from obese adipose tissue actively enhance bone marrow myelopoiesis partly via local activation of the alarmin S100A8/A9, which drives production of IL-1β [84]. Other adipokines have also been shown to govern myelopoiesis, including leptin [85], further corroborating the observation that adipose tissue appears to determine bone marrow function via remote control. It is worth noting that fat residing within the bone marrow is known to express various cytokines and adipokines that change during the development of obesity and type 2 diabetes and might also be involved in the remodelling and activation of bone marrow cell populations [86]. Even though these observations shed some light on the mechanism of action involved, many issues concerning recruitment remain unresolved. In addition to adipose tissue-derived IL-1β, are haematopoietic factors involved in the control of myelopoiesis during obesity as well? Are signals from the adipose tissue also responsible for controlling bone marrow myelopoiesis in lean conditions? And are monocytes phenotypically different when originating from bone marrow during lean vs obese conditions? One line of evidence suggests that monocytes originating from bone marrow of lean or obese animals have a similar migration capacity. Using PKH26 staining to track monocytes, ATM accumulation was found to be enhanced in obese recipient mice compared with lean mice, independently of the origin of donor monocytes from either obese or lean animals [87]. These results imply that adipose tissue-derived signals determine local macrophage numbers. Nonetheless, it has been demonstrated that bone-derived macrophages from HFD-fed mice display an enhanced inflammatory phenotype compared with macrophages derived from bone marrow of lean animals [88]. Moreover, haematopoietic stem cells from obese mice harbour an enhanced capacity to develop into inflammatory CD11c^+^ ATMs after bone marrow transplantation [89]. Together these data suggest that adipose tissue-derived factors determine both the infiltration and differentiation of monocytes and that obesity affects the characteristics of the bone marrow cell population, ultimately translating into phenotypically different monocytes infiltrating lean and obese adipose tissue.

### Local regulation of ATM numbers

In addition to recruitment during obesity, multiple lines of evidence suggest that adipose tissue is equipped with tools allowing for local regulation and proliferation of ATM pools independently of the influx of blood precursors. vAT that is part of human omentum displays enhanced myelopoiesis during acute inflammatory conditions, which is suggestive for in situ production of cell populations in the abdomen [90, 91]. In line with these results, the presence of haematopoietic stem/progenitor cell populations in mouse white adipose tissue has been reported. The capacity of these cells to differentiate into myeloid and lymphoid lineages was similar to that of bone marrow-derived haematopoietic cells [92], yet no evidence currently exists supporting a fundamental role for local myelopoiesis in determining ATM numbers in obese adipose tissue. Another pathway contributing to influx-independent pathways that nourish ATM numbers concerns the involvement of pre-adipocytes that have been assigned with characteristics that closely resemble macrophages. 3T3-L1 pre-adipocytes, a frequently used model to study adipogenesis in vitro, display macrophage-like functions that are lost upon differentiation towards adipocytes [93]. Moreover, pre-adipocytes isolated from adipose tissue carry out many macrophage-like functions including phagocytosis and express macrophage-specific antigens including F4/80, macrophage 1 antigen (Mac-1) and CD45 [94]. Overall, these results imply closely resembling phenotypes of pre-adipocytes and macrophages. When receiving the appropriate signals, pre-adipocytes may efficiently and rapidly adapt and execute ATM functions.

Besides the contribution of local precursors, local proliferation has been held responsible for increasing ATM numbers during obesity [95]. Moreover, ATMs in obese adipose tissue have been postulated to have reduced migration capacity through increased netrin-1 expression that traps them in adipose tissue [96]. Finally, reduced apoptosis of resident ATMs has been suggested to account for enhanced ATM numbers during obesity, independently of monocyte influx [97].

To summarise, the origin of ATMs in lean and obese adipose tissue is still a matter of debate. The presence of specific cell surface markers on ATMs might help us learn more about their origin. Moreover, lineage tracing of ATMs using time-lapse microscopy, cell labelling and/or genetic markers will help us understand the origin of ATMs. On the basis of the currently available data, ATMs appear to be mainly derived from bone marrow-controlled pathways involving adipose-derived signals. Other lines of evidence suggest that local mechanisms may determine macrophage numbers in adipose tissue, including prolonged survival or increased retention of ATMs. Probably, various pathways at multiple levels are at work to set ATM numbers in adipose tissue. Whether the number of ATMs is primarily regulated at the level of the bone marrow or is determined by local factors in the adipose tissue will need further study.

## Drivers of the adipose tissue macrophage phenotype

Both during myelopoiesis and in the circulation, monocytes can be primed by different factors that will eventually contribute to their migration and inflammatory fate after infiltration into the adipose tissue. It is likely that adipose tissue-derived factors will further determine the final ATM phenotype, which may differ between lean and obese adipose tissue. Some important triggers that shape the metabolic, inflammatory and functional traits of ATMs will be discussed below.

As explained earlier, other immune cells residing in the adipose tissue probably shape ATM phenotype as well, but these will not be discussed in this review. The same also applies to various adipokines that are known to be differentially secreted by lean rather than obese adipose tissue. Their role in affecting the phenotype of macrophages has been reviewed elsewhere [98, 99].

### Hypoxia

Both in humans and mice, rapid adipose tissue mass expansion during the development of obesity occurs without a concurrent increase in blood flow towards the tissue [100]. Indeed, animal studies have demonstrated that obese adipose tissue is characterised by hypoxic areas and an increased expression of hypoxia-related genes including *HIF1α* [19, 101–103]. In humans, oxygen levels in the adipose tissue are much harder to measure, and this technical challenge likely contributes to the contradictory results that have been reported so far, ranging from decreased oxygen tension [104, 105], unaltered oxygen tension, and even to increased oxygen tension [106] in obese adipose tissue.

As a result of their profound increase in size upon the development of obesity, adipocytes are generally thought to suffer from hypoxia in obese adipose tissue. Hypoxic conditions in adipocytes promote angiogenesis and fibrotic remodelling and have often been associated with the development of adipose tissue insulin resistance [102, 103, 107].

Only recently has attention been directed to the effects of hypoxic conditions on macrophages in the adipose tissue. In line with adipocytes, ATMs from obese adipose tissue have increased expression levels of hypoxia-related genes [101, 108]. It is not unthinkable that prolonged hypoxia is one of the driving forces behind the proinflammatory phenotype of macrophages in obese adipose tissue. When CD14^+^ cells from human obese vAT are exposed to hypoxic conditions, the secretion of proinflammatory cytokines is enhanced in comparison with culturing under normoxic conditions [70]. Moreover, human macrophages derived from circulating monocytes held under hypoxic conditions show an increased inflammatory response when exposed to the saturated fatty acid (SFA) palmitate [109]. Interestingly, in vivo, M1-like macrophages accumulate more pimonidazole, a hypoxia probe, than do anti-inflammatory macrophages in the adipose tissue of obese mice, and display higher expression levels of hypoxia-related genes including *Hif1α* [101]. Together these data underline a direct link between the hypoxic state of ATMs and their inflammatory phenotype in obese adipose tissue.

Importantly, the response of macrophages to hypoxia might depend on their initial inflammatory state. Indeed, it has been proposed that stimulation of bone marrow-derived macrophages with IL-4 prior to hypoxia exposure decreases the expression of proinflammatory genes, while leaving hypoxia-related gene expression unaltered [101]. Alternatively, differential responses of macrophages to hypoxia could also relate to the presence of two different isoforms of hypoxia-inducible factor (HIF). HIF1α is the most well-known isoform and mediates a shift towards glycolysis, known as the Warburg effect [110]. Inflammatory stimuli are prominent stimulants of HIF1α activity, inducing rapid ATP production independent of oxygen [110, 111]. During hypoxic conditions HIF1α activity is also considered to be crucial to maintain energy levels [100], although other transcription factors probably play an additional role [109]. In addition to fuelling a metabolic switch, HIF1α enhances proinflammatory signalling via IL-1β transcription [110]. The control of IL-1β expression might provide a direct link between HIF1α activity in obese ATMs, either activated via hypoxia or inflammatory signalling, and the presence of insulin resistance.

The isoform HIF2α is also upregulated under low oxygen levels. While HIF1α increases the glycolytic flux and proinflammatory signalling, HIF2α is associated with a more anti-inflammatory macrophage phenotype [112, 113]. Interestingly, in obese adipose tissue HIF2α is predominantly upregulated in M2-like macrophages [108] and peritoneal macrophages overexpressing HIF2α show a blunted increase in TNFα expression on co-culturing with obese adipose tissue [108]. Adipocyte-specific deletion of *Hif2α* has been linked to adipose tissue inflammation, glucose intolerance and insulin resistance [114] but so far no studies specifically targeting HIF2α in macrophages have been conducted.

Overall, the inflammatory state of macrophages might be linked to the relative presence of HIF isoforms that may influence the metabolic and inflammatory response of ATMs under hypoxic conditions. Importantly, in addition to hypoxia, one should not ignore the fact that HIF-dependent gene expression can also be regulated independently of oxygen availability, through the involvement of inflammatory-related signals. In addition to direct effects of hypoxia on macrophages, signals originating from hypoxic adipocytes may also shape the phenotype of ATMs in obese adipose tissue. Indeed, in vitro experiments have revealed that adipocytes exposed to hypoxic conditions release potential stressors that affect macrophage function. Examples include the proinflammatory adipokines IL-6, visfatin, leptin and macrophage migration inhibitory factor (MIF) [100]. Importantly, prolonged exposure to hypoxia might also lead to adipocyte cell death and release of intracellular content which subsequently affects the phenotype of ATMs.

### Adipocyte cell death

In animal studies, cell death in obese adipose tissue has been well established. Dead adipocytes that have lost membrane integrity are surrounded by macrophages that cluster in CLSs and together form proinflammatory, fibrotic lesions [115]. Signals released from dying cells are known to act as strong chemoattractants and affect immune responses [116, 117]. One example is the nuclear protein high-mobility group box 1 (HMGB1), which acts as a danger signal that attracts and activates immune cells [118]. Interestingly, HMGB1 levels are increased in obese adipose tissue and are associated with inflammation [119, 120]. In addition to HMGB1, other cellular proteins with intrinsic inflammatory features are released upon cell death and may skew ATM phenotypes in obese adipose tissue. Uric acid, adenosine, ATP and galectins are among some of these so-called ‘danger signals’ that affect inflammatory macrophage responses [116, 117, 121].

The type of adipocyte cell death occurring in adipose tissue may determine the inflammatory phenotype of macrophages as well. Apoptotic cells induce a predominant anti-inflammatory response, whereas necrotic cells stimulate proinflammatory cytokine secretion [117]. Dead cells that are not effectively cleared are known to turn into secondary necrotic cells releasing their noxious content to induce proinflammatory signalling, autoimmunity and tissue damage [122–125]. Although impaired clearance of dead cells has been causally linked to various chronic inflammatory diseases [29], to date there have been no extensive studies on the contribution of adipocyte death to inflammation during obesity. Some studies have reported necrotic adipocyte cell death in obese adipose tissue, with membrane rupture and release of cell content [9]. However, others postulate programmed necrotic cell death [126], programmed pyroptosis [127] or even apoptosis [128] of adipocytes in obese adipose tissue, making it difficult to draw conclusions on the effect of adipocyte cell death on ATM phenotype. Considering the profound consequence for macrophage phenotype found in other organs, examination of the type of adipocyte cell death occurring in obese adipose tissue, but also during regular cell turnover in lean adipose tissue, is of relevance.

Importantly, adipocyte cell death is unique in the fact that it leads to the release of substantial amounts of lipids and one might hypothesise that prolonged exposure to excess lipids affects ATM phenotype.

### Lipotoxicity

A lipid-rich environment during the development of obesity may represent an important metabolic stressor for ATMs. In general, there are three ways through which ATMs are exposed to lipids; via chylomicrons or VLDLs, by adipocyte lipolysis, or after adipocyte cell death. Hyperlipidaemia seems not to lead to macrophage accumulation in obese adipose tissue [129], indicating that lipids resulting from adipocyte lipolysis and adipocyte cell death represent the main causes of excessive lipid accumulation in ATMs during obesity. However, the exact contribution of adipocyte lipolysis is also somewhat controversial. On the one hand higher basal rates of lipolysis have been observed in adipocytes of obese individuals [130]. In line with this, adipocyte hypertrophy and TNFα exposure, which are both elevated in obese adipose tissue, have been linked to increased adipocyte lipolysis [131–133]. On the other hand, decreased basal lipolytic rates [134] and reduced catecholamine-induced lipolysis by insulin resistant adipocytes in obese adipose tissue [135, 136] have been reported as well, and are indicative of lower lipolysis rates in obese adipose tissue. Nonetheless, although the exact source is still a matter of debate, macrophages in obese adipose tissue are exposed to excess lipids as illustrated by their lipid-laden appearance. Exposure to FAs is known to influence macrophage phenotype profoundly. Importantly, SFAs induce a proinflammatory macrophage phenotype via Toll-like receptor (TLR)-induced NF-kB activation [137]. The TLR-family member 4 (TLR4) translates most of the proinflammatory effects of SFAs that trigger TLR4 activation via binding to its adaptor molecule fetuin-A [138, 139], although some argue that the effects of SFAs are partly TLR-independent [57, 140]. Another source of proinflammatory signalling finds it origin intracellularly, as an increase in intracellular FAs is associated with endoplasmic reticulum (ER) stress and oxidative stress, enhancing proinflammatory signalling and inducing insulin resistance in vivo [141]. Moreover, SFAs and their derivatives, including ceramides, are classified as danger-associated molecular patterns (DAMPs) that are recognised by the NLRP3 inflammasome and lead to IL-1β secretion via caspase 1 activation [142]. However, the response of macrophages upon lipid uptake does not necessarily have to be proinflammatory in nature [143]. Although it has been suggested that internalised palmitate, a very well-known SFA, promotes lipid metabolism and limits inflammation [57], in general only unsaturated FAs, particularly *n*-3 FAs, are recognised for their anti-inflammatory properties [139, 144]. Hence, FA species present in adipocytes may for a large part determine the degree of lipid toxicity in macrophages. Alternatively, one might speculate that the metabolic and inflammatory state of macrophages could also be involved in determining its response to different FAs. Overall, mechanisms by which intracellular FAs determine the inflammatory traits of macrophages are relatively unclear. Importantly, lipid-overloaded macrophages in obese adipose tissue [42] may trigger the production of proinflammatory cytokines such as TNFα and IL-1β, as has been observed in macrophages exposed to FAs in vitro [138, 145–147], and thus contributing to the development of insulin resistance. The type of FAs stored in the adipocyte, the timespan of lipid exposure, the inflammatory phenotype of the macrophage and the presence of various other inflammatory signalling molecules might explain the predominant anti-inflammatory response of ATMs in lean adipose tissue vs the proinflammatory response in obese adipose tissue following lipid exposure.

### Hyperinsulinaemia

Obesity-induced adipose tissue inflammation is associated with systemic insulin resistance and hyperinsulinaemia in obese rodents and humans. Multiple lines of evidence suggest that insulin itself directly affects inflammatory processes. In fact, insulin has been assigned with both anti- and proinflammatory properties. In circulating mononuclear cells of obese individuals, insulin inhibits NF-kB activity, suggesting an anti-inflammatory effect [148]. By contrast, other studies have shown that hyperinsulinaemia promotes proinflammatory responses by affecting T cell regulatory function [149].

It is difficult to pinpoint the sole effects of insulin in vivo during the development of obesity, as changes in many other circulating factors accompany hyperinsulinaemia. However, several lines of evidence suggest that insulin directly contributes to ATM homing. The start of insulin therapy in patients diagnosed with type 2 diabetes has been shown to promote the appearance of macrophages in scAT [150]. Intriguingly, in both humans and mice, circulating insulin positively correlates with inflammatory cytokine expression in the adipose tissue, independently of body weight differences [151]. Recently, a mechanism of action whereby insulin may control propagation of adipose tissue inflammation was uncovered. Even though adipocytes become insensitive to insulin-dependent glucose uptake during obesity, insulin is able to enhance adipocyte *MCP-1* gene expression levels aggravating the influx of monocytes [151].

Insulin resistance has also been shown to negatively affect macrophage function itself, especially in the context of atherosclerotic disease. Although glucose uptake primarily takes place via insulin-independent pathways involving GLUT1, circulating monocytes express the insulin receptor machinery and develop resistance to the effects of insulin. Interestingly, insulin receptor deficiency on macrophages reduces their influx into the adipose tissue and alleviates the development of low-grade inflammation and systemic insulin resistance in mice on an HFD [152]. Functional consequences of insulin resistance include a failure to deal with ER stress, which enhances the risk of macrophage apoptosis and subsequent loss of function [153]. In addition to apoptosis, primary macrophages isolated from *ob*/*ob* mice have a defect in efferocytosis that was associated with defective phosphoinositide (PI)3 kinase activity, whereas cells lacking the insulin receptor were protected [154].

It remains to be determined whether insulin resistance develops in ATMs in obese adipose tissue. Moreover, the functional consequences and possible contributions to adipose tissue inflammation are currently unknown. Another interesting aspect involves the development of insulin resistance in circulating monocytes [155]. It is not unthinkable that insulin resistance in circulating monocytes may promote functional changes leading to altered migration and inflammatory properties. Studies involving monocytes lacking specific parts of the insulin signalling machinery would shed light on the role of this pathway in shaping ATM phenotypes both during lean and obese conditions.

### Hyperglycaemia

Chronic hyperglycaemia is known to induce insulin resistance by mechanisms that may partly be conveyed by an enhanced inflammatory state. Indeed, there is a substantial amount of evidence demonstrating proinflammatory effects of prolonged periods of hyperglycaemia. In humans, hyperglycaemia is associated with inflammation in individuals with diabetes [156]. Similar data has been generated using various animal models where the induction of hyperglycaemia using streptozotocin (STZ)-mediated disruption of pancreatic beta cells drives monocyte recruitment to tissues and promotes inflammation [157]. Other approaches to induce short-term periods of hyperglycaemia, including clamping, trigger proinflammatory responses in adipose tissue of rats as well [158].

In both adipocytes and macrophages, harmful effects of prolonged exposure to high levels of glucose have been reported. In vitro approaches in which adipocytes are cultured in medium with 25 mmol/l glucose for prolonged periods of time revealed increased production of various proinflammatory cytokines including IL-6 [158], and similar observations have been made in immune cells [159]. Moreover, hyperglycaemia has been shown to interfere with IL-4 action to polarise macrophages towards an alternatively activated state illustrated by decreased expression of M2 markers and a reduction in arginase functional activity, compared with cells treated under normoglycaemic conditions [160]. It has been postulated that the induction of reactive oxygen species (ROS) as a consequence of hyperglycaemia evokes inflammatory responses. An important molecular switch that translates the presence of hyperglycaemia into proinflammatory conditions is the thioredoxin-interacting protein (TXNIP). TXNIP expression is enhanced by high levels of glucose and in the presence of diabetes via the transcription factor carbohydrate response element-binding protein (ChREBP) and regulates a variety of processes including redox state and inflammation [161]. Deletion of *Txnip* using small interfering (si)RNA approaches prevents hyperglycaemia-induced ROS generation and the induction of inflammation [162]. Moreover, TXNIP has been linked to inflammasome activation in macrophages [163], a pathway known to contribute to adipose tissue inflammation and insulin resistance [79, 164]. In adipose tissue, glucose-induced activation of TXNIP mediates *IL-1β* mRNA expression levels and intracellular pro-IL-1β accumulation [66].

The consequences of hyperglycaemia may also be conveyed via changes in cellular metabolism leading to alterations in intermediate metabolites. It has been documented that cultured adipocytes exposed to high levels of glucose produce and secrete enhanced quantities of lactate [165]. Interestingly, diabetes is associated with markedly increased lactate production in adipocytes derived from obese adipose tissue [166]. Lactic acid, secreted from tumour cells as a by-product of aerobic or anaerobic glycolysis, has been shown to drive an M2-like polarisation of tumour-associated macrophages [167]. Whether lactic acid secreted by adipocytes may alter ATM phenotype is currently unknown.

High levels of glucose may thus represent an important trigger that activates proinflammatory changes in macrophages, either directly or indirectly. Interestingly, both shorter periods of hyperglycaemia, either during a clamp procedure or postprandially, and chronic periods in the presence of obesity and insulin resistance, appear to induce proinflammatory macrophage characteristics.

## A future challenge: identification of key regulators of macrophage phenotype

Macrophages can be identified via specific transcription factors that control tissue-tailored transcriptional programs and allow the cells to adopt extremely tissue-specific functions. An elegant example is the identification of GATA-6 as master regulator of the peritoneal-specific gene transcriptional program. By comparing the transcriptome of various tissue macrophages including those from lung, liver, adipose tissue and peritoneum [90], the authors identified a set of genes controlled by GATA-6 specifically expressed in peritoneal macrophages. Using a similar approach, we have re-analysed their dataset to pinpoint ATM-specific genes leading to the identification of transforming growth factor (TGF)β and IL-4 as important upstream regulators. The identification of anti-inflammatory upstream regulators in ATMs isolated from lean animals is not surprising, yet it does demonstrate the usefulness of this approach. Comparing transcriptional regulators of ATMs during various conditions may bypass the rather complex quest for responsible triggers, and may help to unravel important pathways and molecules that underlie the distinct metabolic, inflammatory and functional traits of ATMs as a consequence of multiple triggers present in lean vs obese adipose tissue.

**Fig. 1:**
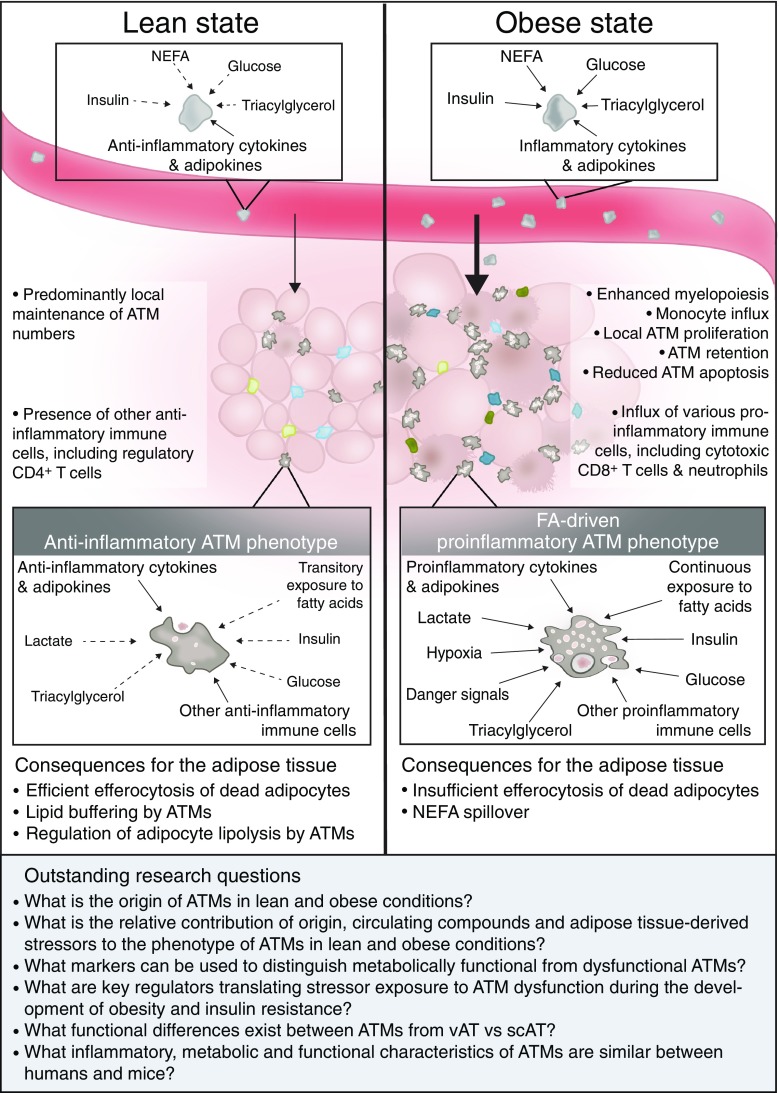
Overview of the different stressors that shape ATM function in lean vs obese adipose tissue

Because of the high versatility of macrophages, which have a phenotype largely dependent on their environment, conclusions solely based on in vitro studies should be taken with caution. Rather, in vivo profiling of macrophage phenotype via several techniques, including metabolomics and transcriptomics, and identification of key regulators of distinct macrophage phenotypes, residing in different adipose tissue depots and/or during the development of obesity and insulin resistance in both humans and mice, will advance our knowledge on adipose tissue biology. Determining a causal relationship between triggers, a switch in the metabolic and inflammatory status of ATMs and ATM function will be an important challenge. This information will be crucial for our understanding of initial triggers that ultimately link obesity to adipose tissue inflammation and the development of insulin resistance, and might provide us with future therapeutic targets. An overview of stressors that may determine the phenotype of ATMs in lean vs obese conditions and the subsequent consequences for adipose tissue function are shown in Fig. 1.

## Limiting adipose tissue inflammation by targeting macrophage metabolism

The presence of adipose tissue inflammation translates the development of obesity into insulin resistance and type 2 diabetes. Macrophages play a key role in maintaining adipose tissue homeostasis, yet fuel adipose tissue inflammation upon the development of obesity.

Current efforts to unravel adipose tissue-specific functions of macrophages have revealed an essential role in lipid buffering for ATMs, alongside their more conventional functions such as clearing cellular debris and participating in tissue immune surveillance to maintain homeostasis. Importantly, both in lean and obese adipose tissue, lipid storage by macrophages prevents spillover into the circulation. However, the metabolically activated macrophage that resides in obese adipose tissue appears to be continuously exposed to an overabundance of lipids. Chronic lipid overloading of ATMs translates into a proinflammatory phenotype and may drive dysfunction of ATMs, ultimately fuelling the inflammatory traits of obese adipose tissue.

This new knowledge might shift intervention approaches away from targeting the inflammatory traits of ATMs, towards targeting their metabolic programming. Interventions aimed at increasing metabolic capacity might be used to reprogram macrophage metabolism, allowing macrophages to cope with metabolic challenges during obesity, in order to maintain adipose tissue homeostasis.

## List of abbreviations

ATM: Adipose tissue macrophage
CCR: C–C chemokine receptor
CLS: Crown-like structure
ER: Endoplasmic reticulum
FA: Fatty acid
HFD: High-fat diet
HIF: Hypoxia-inducible factor
HMGB1: High-mobility group box 1
MCP-1: Monocyte chemoattractant protein-1
ROS: Reactive oxygen species
scAT: Subcutaneous adipose tissue
SFA: Saturated fatty acid
TLR: Toll-like receptor
TXNIP: Thioredoxin-interacting protein
vAT: Visceral adipose tissue

## References

[1] Shoelson SE, Herrero L, Naaz A (2007). Obesity, inflammation, and insulin resistance. Gastroenterology.

[2] Wild S, Roglic G, Green A, Sicree R, King H (2004). Global prevalence of diabetes: estimates for the year 2000 and projections for 2030. Diabetes Care.

[3] Trayhurn P, Wood IS (2004). Adipokines: inflammation and the pleiotropic role of white adipose tissue. Br J Nutr.

[4] Odegaard JI, Chawla A (2008). Mechanisms of macrophage activation in obesity-induced insulin resistance. Nat Clin Pract Endocrinol Metab.

[5] Weisberg SP, McCann D, Desai M, Rosenbaum M, Leibel RL, Ferrante AW (2003). Obesity is associated with macrophage accumulation in adipose tissue. J Clin Invest.

[6] Gericke M, Weyer U, Braune J, Bechmann I, Eilers J (2015). A method for long-term live imaging of tissue macrophages in adipose tissue explants. Am J Physiol Endocrinol Metab.

[7] Lumeng CN, Deyoung SM, Bodzin JL, Saltiel AR (2007). Increased inflammatory properties of adipose tissue macrophages recruited during diet-induced obesity. Diabetes.

[8] Lumeng CN, DelProposto JB, Westcott DJ, Saltiel AR (2008). Phenotypic switching of adipose tissue macrophages with obesity is generated by spatiotemporal differences in macrophage subtypes. Diabetes.

[9] Cinti S, Mitchell G, Barbatelli G (2005). Adipocyte death defines macrophage localization and function in adipose tissue of obese mice and humans. J Lipid Res.

[10] Murano I, Barbatelli G, Parisani V (2008). Dead adipocytes, detected as crown-like structures, are prevalent in visceral fat depots of genetically obese mice. J Lipid Res.

[11] Lee YS, Li P, Huh JY (2011). Inflammation is necessary for long-term but not short-term high-fat diet-induced insulin resistance. Diabetes.

[12] Bigornia SJ, Farb MG, Mott MM (2012). Relation of depot-specific adipose inflammation to insulin resistance in human obesity. Nutr Diabetes.

[13] Xu H, Barnes GT, Yang Q (2003). Chronic inflammation in fat plays a crucial role in the development of obesity-related insulin resistance. J Clin Invest.

[14] Stefanovic-Racic M, Yang X, Turner MS (2012). Dendritic cells promote macrophage infiltration and comprise a substantial proportion of obesity-associated increases in CD11c^+^ cells in adipose tissue and liver. Diabetes.

[15] Liu J, Divoux A, Sun J (2009). Genetic deficiency and pharmacological stabilization of mast cells reduce diet-induced obesity and diabetes in mice. Nat Med.

[16] Talukdar S, da Oh Y, Bandyopadhyay G (2012). Neutrophils mediate insulin resistance in mice fed a high-fat diet through secreted elastase. Nat Med.

[17] Winer DA, Winer S, Shen L (2011). B cells promote insulin resistance through modulation of T cells and production of pathogenic IgG antibodies. Nat Med.

[18] DeFuria J, Belkina AC, Jagannathan-Bogdan M (2013). B cells promote inflammation in obesity and type 2 diabetes through regulation of T cell function and an inflammatory cytokine profile. Proc Natl Acad Sci U S A.

[19] Rausch ME, Weisberg S, Vardhana P, Tortoriello DV (2008). Obesity in C57BL/6J mice is characterized by adipose tissue hypoxia and cytotoxic T cell infiltration. Int J Obes.

[20] Winer S, Chan Y, Paltser G (2009). Normalization of obesity-associated insulin resistance through immunotherapy. Nat Med.

[21] Lynch L, Nowak M, Varghese B (2012). Adipose tissue invariant NKT cells protect against diet-induced obesity and metabolic disorder through regulatory cytokine production. Immunity.

[22] Kintscher U, Hartge M, Hess K (2008). T-lymphocyte infiltration in visceral adipose tissue: a primary event in adipose tissue inflammation and the development of obesity-mediated insulin resistance. Arterioscler Thromb Vasc Biol.

[23] Feuerer M, Herrero L, Cipolletta D (2009). Lean, but not obese, fat is enriched for a unique population of regulatory T cells that affect metabolic parameters. Nat Med.

[24] Sun S, Ji Y, Kersten S, Qi L (2012). Mechanisms of inflammatory responses in obese adipose tissue. Annu Rev Nutr.

[25] Cildir G, Akincilar SC, Tergaonkar V (2013). Chronic adipose tissue inflammation: all immune cells on the stage. Trends Mol Med.

[26] Murray PJ, Wynn TA (2011). Protective and pathogenic functions of macrophage subsets. Nat Rev Immunol.

[27] Lumeng CN, Bodzin JL, Saltiel AR (2007). Obesity induces a phenotypic switch in adipose tissue macrophage polarization. J Clin Invest.

[28] Kerr JF, Wyllie AH, Currie AR (1972). Apoptosis: a basic biological phenomenon with wide-ranging implications in tissue kinetics. Br J Cancer.

[29] Poon IK, Lucas CD, Rossi AG, Ravichandran KS (2014). Apoptotic cell clearance: basic biology and therapeutic potential. Nat Rev Immunol.

[30] Spalding KL, Arner E, Westermark PO (2008). Dynamics of fat cell turnover in humans. Nature.

[31] Strawford A, Antelo F, Christiansen M, Hellerstein MK (2004). Adipose tissue triglyceride turnover, de novo lipogenesis, and cell proliferation in humans measured with ^2^H_2_O. Am J Physiol Endocrinol Metab.

[32] Hirsch J, Batchelor B (1976). Adipose tissue cellularity in human obesity. Clin Endocrinol Metab.

[33] Fischer-Posovszky P, Wang QA, Asterholm IW, Rutkowski JM, Scherer PE (2011). Targeted deletion of adipocytes by apoptosis leads to adipose tissue recruitment of alternatively activated M2 macrophages. Endocrinology.

[34] Lee YH, Petkova AP, Granneman JG (2013). Identification of an adipogenic niche for adipose tissue remodeling and restoration. Cell Metab.

[35] Kosteli A, Sugaru E, Haemmerle G (2010). Weight loss and lipolysis promote a dynamic immune response in murine adipose tissue. J Clin Invest.

[36] Nguyen KD, Qiu Y, Cui X (2011). Alternatively activated macrophages produce catecholamines to sustain adaptive thermogenesis. Nature.

[37] Rao RR, Long JZ, White JP (2014). Meteorin-like is a hormone that regulates immune-adipose interactions to increase beige fat thermogenesis. Cell.

[38] Qiu Y, Nguyen KD, Odegaard JI (2014). Eosinophils and type 2 cytokine signaling in macrophages orchestrate development of functional beige fat. Cell.

[39] Liu PS, Lin YW, Lee B, McCrady-Spitzer SK, Levine JA, Wei LN (2014). Reducing RIP140 expression in macrophage alters ATM infiltration, facilitates white adipose tissue browning, and prevents high-fat diet-induced insulin resistance. Diabetes.

[40] Hui X, Gu P, Zhang J (2015). Adiponectin enhances cold-induced browning of subcutaneous adipose tissue via promoting M2 macrophage proliferation. Cell Metab.

[41] Xu X, Grijalva A, Skowronski A, van Eijk M, Serlie MJ, Ferrante AW (2013). Obesity activates a program of lysosomal-dependent lipid metabolism in adipose tissue macrophages independently of classic activation. Cell Metab.

[42] Shapiro H, Pecht T, Shaco-Levy R (2013). Adipose tissue foam cells are present in human obesity. J Clin Endocrinol Metab.

[43] Nishimura S, Manabe I, Nagasaki M (2007). Adipogenesis in obesity requires close interplay between differentiating adipocytes, stromal cells, and blood vessels. Diabetes.

[44] Cho CH, Koh YJ, Han J (2007). Angiogenic role of LYVE-1-positive macrophages in adipose tissue. Circ Res.

[45] Wernstedt Asterholm I, Tao C, Morley TS (2014). Adipocyte inflammation is essential for healthy adipose tissue expansion and remodeling. Cell Metab.

[46] Lacasa D, Taleb S, Keophiphath M, Miranville A, Clement K (2007). Macrophage-secreted factors impair human adipogenesis: involvement of proinflammatory state in preadipocytes. Endocrinology.

[47] Constant VA, Gagnon A, Yarmo M, Sorisky A (2008). The antiadipogenic effect of macrophage-conditioned medium depends on ERK1/2 activation. Metab Clin Exp.

[48] Maumus M, Sengenes C, Decaunes P (2008). Evidence of in situ proliferation of adult adipose tissue-derived progenitor cells: influence of fat mass microenvironment and growth. J Clin Endocrinol Metab.

[49] Zaragosi LE, Wdziekonski B, Villageois P (2010). Activin a plays a critical role in proliferation and differentiation of human adipose progenitors. Diabetes.

[50] Bilkovski R, Schulte DM, Oberhauser F (2011). Adipose tissue macrophages inhibit adipogenesis of mesenchymal precursor cells via wnt-5a in humans. Int J Obes.

[51] Hossain P, Kawar B, El Nahas M (2007). Obesity and diabetes in the developing world—a growing challenge. N Engl J Med.

[52] MacDougald OA, Mandrup S (2002). Adipogenesis: forces that tip the scales. Trends Endocrinol Metab.

[53] Osborn O, Olefsky JM (2012). The cellular and signaling networks linking the immune system and metabolism in disease. Nat Med.

[54] Heilbronn LK, Campbell LV (2008). Adipose tissue macrophages, low grade inflammation and insulin resistance in human obesity. Curr Pharm Des.

[55] Zeyda M, Gollinger K, Kriehuber E, Kiefer FW, Neuhofer A, Stulnig TM (2010). Newly identified adipose tissue macrophage populations in obesity with distinct chemokine and chemokine receptor expression. Int J Obes.

[56] Li P, Lu M, Nguyen MT (2010). Functional heterogeneity of CD11c-positive adipose tissue macrophages in diet-induced obese mice. J Biol Chem.

[57] Kratz M, Coats BR, Hisert KB (2014). Metabolic dysfunction drives a mechanistically distinct proinflammatory phenotype in adipose tissue macrophages. Cell Metab.

[58] Zeyda M, Farmer D, Todoric J (2007). Human adipose tissue macrophages are of an anti-inflammatory phenotype but capable of excessive pro-inflammatory mediator production. Int J Obes.

[59] Bourlier V, Zakaroff-Girard A, Miranville A (2008). Remodeling phenotype of human subcutaneous adipose tissue macrophages. Circulation.

[60] Wentworth JM, Naselli G, Brown WA (2010). Pro-inflammatory CD11c^+^CD206^+^ adipose tissue macrophages are associated with insulin resistance in human obesity. Diabetes.

[61] Fjeldborg K, Pedersen SB, Moller HJ, Christiansen T, Bennetzen M, Richelsen B (2014). Human adipose tissue macrophages are enhanced but changed to an anti-inflammatory profile in obesity. J Immunol Res.

[62] Cancello R, Tordjman J, Poitou C (2006). Increased infiltration of macrophages in omental adipose tissue is associated with marked hepatic lesions in morbid human obesity. Diabetes.

[63] Makkonen J, Westerbacka J, Kolak M (2007). Increased expression of the macrophage markers and of 11β-HSD-1 in subcutaneous adipose tissue, but not in cultured monocyte-derived macrophages, is associated with liver fat in human obesity. Int J Obes.

[64] Harman-Boehm I, Bluher M, Redel H (2007). Macrophage infiltration into omental versus subcutaneous fat across different populations: effect of regional adiposity and the comorbidities of obesity. J Clin Endocrinol Metab.

[65] Fox CS, Massaro JM, Hoffmann U (2007). Abdominal visceral and subcutaneous adipose tissue compartments: association with metabolic risk factors in the Framingham Heart Study. Circulation.

[66] Koenen TB, Stienstra R, van Tits LJ (2011). The inflammasome and caspase-1 activation: a new mechanism underlying increased inflammatory activity in human visceral adipose tissue. Endocrinology.

[67] O’Rourke RW, Metcalf MD, White AE (2009). Depot-specific differences in inflammatory mediators and a role for NK cells and IFN-γ in inflammation in human adipose tissue. Int J Obes.

[68] Berry DC, Stenesen D, Zeve D, Graff JM (2013). The developmental origins of adipose tissue. Development.

[69] Wajchenberg BL (2000). Subcutaneous and visceral adipose tissue: their relation to the metabolic syndrome. Endocr Rev.

[70] O’Rourke RW, White AE, Metcalf MD (2011). Hypoxia-induced inflammatory cytokine secretion in human adipose tissue stromovascular cells. Diabetologia.

[71] Spoto B, Di Betta E, Mattace-Raso F (2014). Pro- and anti-inflammatory cytokine gene expression in subcutaneous and visceral fat in severe obesity. Nutr Metab Cardiovasc Dis.

[72] Gautier EL, Yvan-Charvet L (2014). Understanding macrophage diversity at the ontogenic and transcriptomic levels. Immunol Rev.

[73] Davies LC, Jenkins SJ, Allen JE, Taylor PR (2013). Tissue-resident macrophages. Nat Immunol.

[74] Epelman S, Lavine KJ, Beaudin AE (2014). Embryonic and adult-derived resident cardiac macrophages are maintained through distinct mechanisms at steady state and during inflammation. Immunity.

[75] Arner E, Mejhert N, Kulyte A (2012). Adipose tissue microRNAs as regulators of CCL2 production in human obesity. Diabetes.

[76] Kamei N, Tobe K, Suzuki R (2006). Overexpression of monocyte chemoattractant protein-1 in adipose tissues causes macrophage recruitment and insulin resistance. J Biol Chem.

[77] Krinninger P, Ensenauer R, Ehlers K (2014). Peripheral monocytes of obese women display increased chemokine receptor expression and migration capacity. J Clin Endocrinol Metab.

[78] Nguyen MT, Favelyukis S, Nguyen AK (2007). A subpopulation of macrophages infiltrates hypertrophic adipose tissue and is activated by free fatty acids via Toll-like receptors 2 and 4 and JNK-dependent pathways. J Biol Chem.

[79] Vandanmagsar B, Youm YH, Ravussin A (2011). The NLRP3 inflammasome instigates obesity-induced inflammation and insulin resistance. Nat Med.

[80] Gao D, Madi M, Ding C (2014). Interleukin-1β mediates macrophage-induced impairment of insulin signaling in human primary adipocytes. Am J Physiol Endocrinol Metab.

[81] Kanda H, Tateya S, Tamori Y (2006). MCP-1 contributes to macrophage infiltration into adipose tissue, insulin resistance, and hepatic steatosis in obesity. J Clin Invest.

[82] Kitade H, Sawamoto K, Nagashimada M (2012). CCR5 plays a critical role in obesity-induced adipose tissue inflammation and insulin resistance by regulating both macrophage recruitment and M1/M2 status. Diabetes.

[83] Koh YJ, Kang S, Lee HJ (2007). Bone marrow-derived circulating progenitor cells fail to transdifferentiate into adipocytes in adult adipose tissues in mice. J Clin Invest.

[84] Nagareddy PR, Kraakman M, Masters SL (2014). Adipose tissue macrophages promote myelopoiesis and monocytosis in obesity. Cell Metab.

[85] Laharrague P, Larrouy D, Fontanilles AM (1998). High expression of leptin by human bone marrow adipocytes in primary culture. FASEB J.

[86] Krings A, Rahman S, Huang S, Lu Y, Czernik PJ, Lecka-Czernik B (2012). Bone marrow fat has brown adipose tissue characteristics, which are attenuated with aging and diabetes. Bone.

[87] Oh DY, Morinaga H, Talukdar S, Bae EJ, Olefsky JM (2012). Increased macrophage migration into adipose tissue in obese mice. Diabetes.

[88] Chan KL, Pillon NJ, Sivaloganathan DM (2015). Palmitoleate reverses high fat-induced proinflammatory macrophage polarization via AMP-activated protein kinase (AMPK). J Biol Chem.

[89] Singer K, DelProposto J, Morris DL (2014). Diet-induced obesity promotes myelopoiesis in hematopoietic stem cells. Mol Metab.

[90] Okabe Y, Medzhitov R (2014). Tissue-specific signals control reversible program of localization and functional polarization of macrophages. Cell.

[91] Pinho Mde F, Hurtado SP, El-Cheikh MC, Rossi MI, Dutra HS, Borojevic R (2002). Myelopoiesis in the omentum of normal mice and during abdominal inflammatory processes. Cell Tissue Res.

[92] Poglio S, De Toni F, Lewandowski D (2012). In situ production of innate immune cells in murine white adipose tissue. Blood.

[93] Cousin B, Munoz O, Andre M (1999). A role for preadipocytes as macrophage-like cells. FASEB J.

[94] Charriere G, Cousin B, Arnaud E (2003). Preadipocyte conversion to macrophage. Evidence of plasticity. J Biol Chem.

[95] Amano SU, Cohen JL, Vangala P (2014). Local proliferation of macrophages contributes to obesity-associated adipose tissue inflammation. Cell Metab.

[96] Ramkhelawon B, Hennessy EJ, Menager M (2014). Netrin-1 promotes adipose tissue macrophage retention and insulin resistance in obesity. Nat Med.

[97] Hill AA, Anderson-Baucum EK, Kennedy AJ, Webb CD, Yull FE, Hasty AH (2015). Activation of NF-κB drives the enhanced survival of adipose tissue macrophages in an obesogenic environment. Mol Metab.

[98] Barnes MA, Carson MJ, Nair MG (2015). Non-traditional cytokines: how catecholamines and adipokines influence macrophages in immunity, metabolism and the central nervous system. Cytokine.

[99] Bai Y, Sun Q (2015). Macrophage recruitment in obese adipose tissue. Obes Rev.

[100] Trayhurn P (2013). Hypoxia and adipose tissue function and dysfunction in obesity. Physiol Rev.

[101] Fujisaka S, Usui I, Ikutani M (2013). Adipose tissue hypoxia induces inflammatory M1 polarity of macrophages in an HIF-1α-dependent and HIF-1α-independent manner in obese mice. Diabetologia.

[102] Hosogai N, Fukuhara A, Oshima K (2007). Adipose tissue hypoxia in obesity and its impact on adipocytokine dysregulation. Diabetes.

[103] Ye J, Gao Z, Yin J, He Q (2007). Hypoxia is a potential risk factor for chronic inflammation and adiponectin reduction in adipose tissue of *ob/ob* and dietary obese mice. Am J Physiol Endocrinol Metab.

[104] Pasarica M, Sereda OR, Redman LM (2009). Reduced adipose tissue oxygenation in human obesity: evidence for rarefaction, macrophage chemotaxis, and inflammation without an angiogenic response. Diabetes.

[105] Kabon B, Nagele A, Reddy D (2004). Obesity decreases perioperative tissue oxygenation. Anesthesiology.

[106] Goossens GH, Bizzarri A, Venteclef N (2011). Increased adipose tissue oxygen tension in obese compared with lean men is accompanied by insulin resistance, impaired adipose tissue capillarization, and inflammation. Circulation.

[107] Halberg N, Khan T, Trujillo ME (2009). Hypoxia-inducible factor 1α induces fibrosis and insulin resistance in white adipose tissue. Mol Cell Biol.

[108] Choe SS, Shin KC, Ka S, Lee YK, Chun JS, Kim JB (2014). Macrophage HIF-2α ameliorates adipose tissue inflammation and insulin resistance in obesity. Diabetes.

[109] Snodgrass RG, Boss M, Zezina E (2016). Hypoxia potentiates palmitate-induced proinflammatory activation of primary human macrophages. J Biol Chem.

[110] Tannahill GM, Curtis AM, Adamik J (2013). Succinate is an inflammatory signal that induces IL-1β through HIF-1α. Nature.

[111] Haddad JJ, Harb HL (2005). Cytokines and the regulation of hypoxia-inducible factor (HIF)-1α. Int Immunopharmacol.

[112] Fang HY, Hughes R, Murdoch C (2009). Hypoxia-inducible factors 1 and 2 are important transcriptional effectors in primary macrophages experiencing hypoxia. Blood.

[113] Takeda N, O’Dea EL, Doedens A (2010). Differential activation and antagonistic function of HIF-α isoforms in macrophages are essential for NO homeostasis. Genes Dev.

[114] Lee YS, Kim JW, Osborne O (2014). Increased adipocyte O_2_ consumption triggers HIF-1α, causing inflammation and insulin resistance in obesity. Cell.

[115] Divoux A, Tordjman J, Lacasa D (2010). Fibrosis in human adipose tissue: composition, distribution, and link with lipid metabolism and fat mass loss. Diabetes.

[116] Kimura T, Kobayashi S, Hanihara-Tatsuzawa F, Sayama A, MaruYama T, Muta T (2014). Responses of macrophages to the danger signals released from necrotic cells. Int Immunol.

[117] Rock KL, Lai JJ, Kono H (2011). Innate and adaptive immune responses to cell death. Immunol Rev.

[118] Yang H, Tracey KJ (2005). High mobility group box 1 (HMGB1). Crit Care Med.

[119] Gunasekaran MK, Viranaicken W, Girard AC (2013). Inflammation triggers high mobility group box 1 (HMGB1) secretion in adipose tissue, a potential link to obesity. Cytokine.

[120] Guzman-Ruiz R, Ortega F, Rodriguez A (2014). Alarmin high-mobility group B1 (HMGB1) is regulated in human adipocytes in insulin resistance and influences insulin secretion in beta-cells. Int J Obes.

[121] Rock KL, Kono H (2008). The inflammatory response to cell death. Annu Rev Pathol.

[122] Voll RE, Herrmann M, Roth EA, Stach C, Kalden JR, Girkontaite I (1997). Immunosuppressive effects of apoptotic cells. Nature.

[123] Iyer SS, Pulskens WP, Sadler JJ (2009). Necrotic cells trigger a sterile inflammatory response through the Nlrp3 inflammasome. Proc Natl Acad Sci U S A.

[124] Erwig LP, Henson PM (2008). Clearance of apoptotic cells by phagocytes. Cell Death Differ.

[125] Korns D, Frasch SC, Fernandez-Boyanapalli R, Henson PM, Bratton DL (2011). Modulation of macrophage efferocytosis in inflammation. Front Immunol.

[126] Feng D, Tang Y, Kwon H (2011). High-fat diet-induced adipocyte cell death occurs through a cyclophilin D intrinsic signaling pathway independent of adipose tissue inflammation. Diabetes.

[127] Giordano A, Murano I, Mondini E (2013). Obese adipocytes show ultrastructural features of stressed cells and die of pyroptosis. J Lipid Res.

[128] Alkhouri N, Gornicka A, Berk MP (2010). Adipocyte apoptosis, a link between obesity, insulin resistance, and hepatic steatosis. J Biol Chem.

[129] Coenen KR, Gruen ML, Chait A, Hasty AH (2007). Diet-induced increases in adiposity, but not plasma lipids, promote macrophage infiltration into white adipose tissue. Diabetes.

[130] Langin D, Dicker A, Tavernier G (2005). Adipocyte lipases and defect of lipolysis in human obesity. Diabetes.

[131] Bjorntorp P, Sjostrom L (1972). The composition and metabolism in vitro of adipose tissue fat cells of different sizes. Eur J Clin Investig.

[132] Laurencikiene J, Skurk T, Kulyte A (2011). Regulation of lipolysis in small and large fat cells of the same subject. J Clin Endocrinol Metab.

[133] Langin D, Arner P (2006). Importance of TNFα and neutral lipases in human adipose tissue lipolysis. Trends Endocrinol Metab.

[134] Cifuentes M, Albala C, Rojas CV (2008). Differences in lipogenesis and lipolysis in obese and non-obese adult human adipocytes. Biol Res.

[135] Arner P, Bernard S, Salehpour M (2011). Dynamics of human adipose lipid turnover in health and metabolic disease. Nature.

[136] Ryden M, Andersson DP, Bernard S, Spalding K, Arner P (2013). Adipocyte triglyceride turnover and lipolysis in lean and overweight subjects. J Lipid Res.

[137] Wei Y, Wang D, Topczewski F, Pagliassotti MJ (2006). Saturated fatty acids induce endoplasmic reticulum stress and apoptosis independently of ceramide in liver cells. Am J Physiol Endocrinol Metab.

[138] Pal D, Dasgupta S, Kundu R (2012). Fetuin-A acts as an endogenous ligand of TLR4 to promote lipid-induced insulin resistance. Nat Med.

[139] Chait A, Kim F (2010). Saturated fatty acids and inflammation: who pays the toll?. Arterioscler Thromb Vasc Biol.

[140] Anderson EK, Hill AA, Hasty AH (2012). Stearic acid accumulation in macrophages induces Toll-like receptor 4/2-independent inflammation leading to endoplasmic reticulum stress-mediated apoptosis. Arterioscler Thromb Vasc Biol.

[141] Boden G, Duan X, Homko C (2008). Increase in endoplasmic reticulum stress-related proteins and genes in adipose tissue of obese, insulin-resistant individuals. Diabetes.

[142] Lamkanfi M, Mueller JL, Vitari AC (2009). Glyburide inhibits the Cryopyrin/Nalp3 inflammasome. J Cell Biol.

[143] Caspar-Bauguil S, Kolditz CI, Lefort C (2015). Fatty acids from fat cell lipolysis do not activate an inflammatory response but are stored as triacylglycerols in adipose tissue macrophages. Diabetologia.

[144] Choque B, Catheline D, Rioux V, Legrand P (2014). Linoleic acid: between doubts and certainties. Biochimie.

[145] Zhang J, Gao Z, Yin J, Quon MJ, Ye J (2008). S6K directly phosphorylates IRS-1 on Ser-270 to promote insulin resistance in response to TNF-α signaling through IKK2. J Biol Chem.

[146] Jager J, Gremeaux T, Cormont M, Le Marchand-Brustel Y, Tanti JF (2007). Interleukin-1β-induced insulin resistance in adipocytes through down-regulation of insulin receptor substrate-1 expression. Endocrinology.

[147] Olholm J, Paulsen SK, Cullberg KB, Richelsen B, Pedersen SB (2010). Anti-inflammatory effect of resveratrol on adipokine expression and secretion in human adipose tissue explants. Int J Obes.

[148] Dandona P, Aljada A, Mohanty P (2001). Insulin inhibits intranuclear nuclear factor κB and stimulates IκB in mononuclear cells in obese subjects: evidence for an anti-inflammatory effect?. J Clin Endocrinol Metab.

[149] Han JM, Patterson SJ, Speck M, Ehses JA, Levings MK (2014). Insulin inhibits IL-10-mediated regulatory T cell function: implications for obesity. J Immunol.

[150] Jansen HJ, Stienstra R, van Diepen JA (2013). Start of insulin therapy in patients with type 2 diabetes mellitus promotes the influx of macrophages into subcutaneous adipose tissue. Diabetologia.

[151] Pedersen DJ, Guilherme A, Danai LV (2015). A major role of insulin in promoting obesity-associated adipose tissue inflammation. Mol Metab.

[152] Mauer J, Chaurasia B, Plum L (2010). Myeloid cell-restricted insulin receptor deficiency protects against obesity-induced inflammation and systemic insulin resistance. PLoS Genet.

[153] Han S, Liang CP, DeVries-Seimon T (2006). Macrophage insulin receptor deficiency increases ER stress-induced apoptosis and necrotic core formation in advanced atherosclerotic lesions. Cell Metab.

[154] Tabas I, Tall A, Accili D (2010). The impact of macrophage insulin resistance on advanced atherosclerotic plaque progression. Circ Res.

[155] Olefsky JM, Reaven GM (1976). Insulin binding to monocytes and total mononuclear leukocytes from normal and diabetic patients. J Clin Endocrinol Metab.

[156] de Rekeneire N, Peila R, Ding J (2006). Diabetes, hyperglycemia, and inflammation in older individuals: the health, aging and body composition study. Diabetes Care.

[157] Venneri MA, Giannetta E, Panio G (2015). Chronic inhibition of PDE5 limits pro-inflammatory monocyte–macrophage polarization in streptozotocin-induced diabetic mice. PLoS One.

[158] Lin Y, Berg AH, Iyengar P (2005). The hyperglycemia-induced inflammatory response in adipocytes: the role of reactive oxygen species. J Biol Chem.

[159] Morohoshi M, Fujisawa K, Uchimura I, Numano F (1996). Glucose-dependent interleukin 6 and tumor necrosis factor production by human peripheral blood monocytes in vitro. Diabetes.

[160] Parathath S, Grauer L, Huang LS (2011). Diabetes adversely affects macrophages during atherosclerotic plaque regression in mice. Diabetes.

[161] Chen J, Jing G, Xu G, Shalev A (2014). Thioredoxin-interacting protein stimulates its own expression via a positive feedback loop. Mol Endocrinol.

[162] Devi TS, Lee I, Huttemann M, Kumar A, Nantwi KD, Singh LP (2012). TXNIP links innate host defense mechanisms to oxidative stress and inflammation in retinal Muller glia under chronic hyperglycemia: implications for diabetic retinopathy. Exp Diabetes Res.

[163] Park YJ, Yoon SJ, Suh HW (2013). TXNIP deficiency exacerbates endotoxic shock via the induction of excessive nitric oxide synthesis. PLoS Pathog.

[164] Stienstra R, Joosten LA, Koenen T (2010). The inflammasome-mediated caspase-1 activation controls adipocyte differentiation and insulin sensitivity. Cell Metab.

[165] Sabater D, Arriaran S, Romero Mdel M (2014). Cultured 3T3L1 adipocytes dispose of excess medium glucose as lactate under abundant oxygen availability. Sci Rep.

[166] DiGirolamo M, Newby FD, Lovejoy J (1992). Lactate production in adipose tissue: a regulated function with extra-adipose implications. FASEB J.

[167] Colegio OR, Chu NQ, Szabo AL (2014). Functional polarization of tumour-associated macrophages by tumour-derived lactic acid. Nature.

